# Supplementary material for: “They Just Need to Come Down a Little Bit to Your Level”: A Qualitative Study of Parents’ Views and Experiences of Early Life Interventions to Promote Healthy Growth and Associated Behaviours
Source: Int J Environ Res Public Health. 2020 May 21;17(10):3605. doi: 10.3390/ijerph17103605 (PMC7277501; doi:10.3390/ijerph17103605)
Supplement: Supplementary file 1 [file ijerph-17-03605-s001.zip › SupplementaryMaterials/AdditionalFile2_MaternalandInfantCareinIreland.docx]

**Additional File 2 Maternal and Infant Care in Ireland**

Women in Ireland are entitled to maternity care under the Maternity and Infant Scheme which provides an agreed programme of care to all expectant mothers who are ordinarily resident in Ireland (1). This service is provided by a family doctor (GP) of their choosing and a hospital obstetrician (Table S1).

**Table S1 Maternal and child health services available to women in Ireland**

| **Service type** | **Description** |
| --- | --- |
| **Consultant led service** | Service provided in a maternity hospital/unit by a multidisciplinary team led by a consultant obstetrician (18.1% of live births in 2016 (2)^a^) |
| **Combined care** | Under the Maternity and Infant Care Scheme, care is shared between the GP and the hospital / DOMINO services. (81.6% of live births in 2016 (2)) |
| **Midwife-led units**^a^ | Available to low risk women, where the service is co-located with a consultant-led unit. The service is planned, managed, coordinated and delivered by midwives and covers the antenatal, intrapartum and postnatal periods. Care is delivered in the community and in an alongside midwife-led unit. |
| **DOMINO (Domiciliary In and Out)^a^** | Service generally provided by a team of hospital based community midwives who care for women throughout pregnancy, birth and during the postnatal period. Antenatal appointments can take place either in the hospital or in a community setting. The woman generally transfers home within 12-24 hours after the birth. The community midwife continues to look after mother and baby for the first few days at home. 2,297 DOMINO births were recorded in 2014, accounting for 3.35% of total births. |
| **Early Transfer Home Scheme^a^** | A scheme available in a number of hospitals to facilitate mothers who wish to leave hospital within a few hours after giving birth. Postnatal care is provided by a team of community midwives in the woman’s home. |
| **Home births^a^** | The National Maternity Hospital and University Hospital Waterford offer a very limited home birth service to low risk women. In addition, the HSE facilitates a home birth service through self-employed community midwives (SECM). Approximately 20 SECM have signed Memoranda of Understanding (MOU) with the HSE to provide planned home birth services to eligible women. SECMs are bound by the terms of the MOU and are indemnified under the Clinical Indemnity Scheme operated by the State Claims Agency. The SECM is the primary carer for the woman throughout her pregnancy and for up to 14 days postnatally. Home births account for approximately 0.2% of births in Ireland. |

Source: (3)

^a^Deliveries under hospital schemes (such as domino, early transfer home, planned community midwives, and midwifery-led units) are treated as hospital births as the current birth notification form does not differentiate between different hospital schemes].

**Postnatal care** is provided to all women; however the care is provided in different settings and for a different duration, depending on the model of care. For women birthing in hospitals, postnatal care is provided within the hospital setting. The average length of hospital stay is dictated by the type and complexity of the delivery. The average postnatal length of stay in Irish maternity hospitals/units was 3.4 days in 2016 (2). Women who receive their care in midwifery led units, the DOMINO or Early Transfer Home services, receive postnatal care for the first few days at home. This care is provided as an outreach service by hospital midwives. However, in the main, postnatal care is provided by public health nurses (PHNs) who visit mother and baby at home soon after their discharge from hospital. PHNs visit all mothers and their infants within 72 hours of hospital discharge. They have a key role in supporting child health, families and new babies, screening for postnatal depression, providing breast feeding support and checking the baby’s development, amongst other services. Women who avail of the Maternity and Infant Care Scheme attend their GP for the 6 week postnatal check, and bring their baby to the GP for a 2 week and 6 week check. All infants are entitled to free GP care under the Under 6s GP Visit Card Scheme, which includes free GP assessments at age 2 and 5.

**Table S2 No. of contacts under the Universal Child Health and Wellbeing Programme from pregnancy to a child’s second birthday**

| **Timeline** | **Maternity and Infant Scheme** | | **GP – child only** | | **HSE Child Health, Screening and Surveillance Service** |
| --- | --- | --- | --- | --- | --- |
|  | **Hospital** | **GP^d^** | **Immunisations** | **Under 6s GP contract** |  |
| **Antenatal** | | | | | |
| **Before 12 weeks** |  | 1 |  |  |  |
| **Before 20 weeks** | 1 |  |  |  |  |
| **24 weeks** |  | 1 |  |  |  |
| **28 weeks** | 1 (1^st^ pregnancy) | 1 (except in case of 1^st^ pregnancy) |  |  |  |
| **30 weeks** |  | 1 |  |  |  |
| **32 weeks** | 1 |  |  |  |  |
| **34 weeks** |  | 1 |  |  |  |
| **36 weeks** | 1 |  |  |  |  |
| **37 weeks** |  | 1 |  |  |  |
| **38 weeks** | 1 |  |  |  |  |
| **39 weeks** |  | 1 |  |  |  |
| **40 weeks** | 1 |  |  |  |  |
| **Postnatal** | | | | | |
| **Birth** | 1 ^a,b,c^ |  |  |  |  |
| **Primary visit within 72 hours** |  |  |  |  | 1 ^b,c^ |
| **2 weeks** |  | 1 |  |  |  |
| **6 weeks** |  | 1 (mother & baby) |  |  |  |
| **2 months** |  |  | 1 |  |  |
| **3 months** |  |  |  |  | 1 |
| **4 months** |  |  | 1 |  |  |
| **6 months** |  |  | 1 |  |  |
| **7-9 months** |  |  |  |  | 1 |
| **12 months** |  |  | 1 |  |  |
| **13 months** |  |  | 1 |  |  |
| **18-24 months** |  |  |  |  | 1 |
| **24 months** |  |  |  | 1 |  |
| **Total (25)** | **6/7** | **8/9** | **5** | **1** | **4** |

**Notes:** ^a^ Neonatal Examination and Hearing Screening. ^b^ Newborn Blood Spot Screening can be done in hospital or by PHN. ^c^ BCG. ^d^ If a woman has a significant illness, e.g. diabetes or hypertension, she may have up to 5 additional visits to the GP.

**References**

1. Health Service Executive. Maternity and Infant Care Scheme 2018. Available from: <https://www.hse.ie/eng/services/list/3/maternity/combinedcare.html>.

2. Healthcare Pricing Office. Perinatal Statistics Report 2016. Dublin: Healthcare Pricing Office; 2018.

3. Department of Health. Creating a Better Future Together: National Maternity Strategy 2016-2026. Dublin: Government Publications; 2016.
