# Supplementary material for: “They Just Need to Come Down a Little Bit to Your Level”: A Qualitative Study of Parents’ Views and Experiences of Early Life Interventions to Promote Healthy Growth and Associated Behaviours
Source: Int J Environ Res Public Health. 2020 May 21;17(10):3605. doi: 10.3390/ijerph17103605 (PMC7277501; doi:10.3390/ijerph17103605)
Supplement: Supplementary file 1 [file ijerph-17-03605-s001.zip › SupplementaryMaterials/AdditionalFile3_InterviewGuide.docx]

**Additional File 3 Interview Guide**

**Parents’ views on healthy growth in young children**

**Parent Interview Guide**

| **Study aim:**  To elicit parents’ views on obesity prevention and specifically health professional-delivered obesity prevention interventions targeting children under the age of two.  **Study objectives**   1. To explore perceptions of a good and/or appropriate size (healthy growth) for babies and toddlers up to the age of two 2. To explore parents’ understanding of healthy growth and associated behaviours 3. To establish the perceived importance of weight in young children, how parents gauge it and if/how they think it can be managed 4. To explore parents’ views about childhood obesity, interventions to prevent childhood obesity / promote healthy growth, and interventions delivered by health professionals in particular 5. To examine variations in all the above, if any, by socio-economic background and gender/parental role. |
| --- |

**Briefing**

- Thank you very much for agreeing to take part in the study, I really appreciate you giving up your time to talk with me.
- My name is Marita Hennessy and I am a PhD student within the Health Behaviour Change Research Group in the School of Psychology at NUI Galway.
- As described in the Participant Information Sheet, we are carrying out these interviews to get a better understanding of what parents think about healthy growth in young children and how this can be best supported. We want to use the information to inform the development of supports/programmes.
- I’m really interested to hear your views. There are no right or wrong answers.
- If at any time during the interview you do not wish to answer a question that’s okay, just tell me and we will move on. [This may be particularly relevant if a child is present].
- I would like to record our conversation to allow me to give you my full attention during the interview. The recording will be typed out, but everything you say will be in strict confidence and reported anonymously (unless you disclose something that is identified as a risk to a child, in this case I must report this in line with child protection guidelines). This means that your name and any names or places you mention will be taken out and replaced, so that if someone read your interview they would not know who you are.
- If, at any stage, you wish to stop the audio recorder, or the interview, please let me know.
- Do you have any questions you would like to ask before we start?

**A. Introduction**

- Tell me a bit about yourself and your family (will have completed background survey so will have details) and your baby/toddler in particular
  - Baby’s age
  - Any other children
  - Who else cares for the child/helps them with their child?

**B. Importance of issue**

- We hear a lot in the media nowadays about childhood obesity, what do you think about this issue?
- How do you know if a child aged under two is a healthy weight?
- Is it possible for a baby or toddler to be overweight?
  - What might the consequences of a child being overweight now, or for the future be, if any?
- How much do you think that an infant’s weight needs to be monitored?
  - Are there things you can do to manage an infant’s weight?
  - Is it possible to manage an infant’s weight – to maintain a healthy weight or to manage it if it becomes an issue? If yes, how?

**C. Understanding of behaviours associated with healthy growth**

- What do think influences the size and growth of babies and toddlers?
  - [Open Q first => cue cards if not forthcoming]
  - Your weight when you became pregnant
  - The amount of weight you gain during your pregnancy?
  - If you smoke during pregnancy?
  - A baby’s birth weight?
  - How quickly a baby gains weight during the first few months of life?
  - How and what you feed your baby (breastfeeding/formula feeding; when/how you introduce your baby to solid foods)?
  - How active or inactive a baby is?
  - How much sleep your baby gets?
  - Anything else?
- What do you think are the most important influences?

**D. Experience - pregnancy**

- Did anyone discuss your weight before you became pregnant with you? Can you tell me about this?
- Thinking about your pregnancy - was your weight gain and/or the weight of your baby discussed with you?
  - When? By whom? Where?
  - Did you raise the issue or did someone else?
  - What did you think about this? [Probe various mandatory checks/visits].

**E. General weight perceptions / Experience – post-partum**

- Can you tell me about your baby’s size at birth? What did you think about this?
- Describe your baby’s growth during the first few months of life?
  - What are your thoughts about this?
- What do you think about your baby/toddler’s weight now?
  - As compared to his/her siblings, cousins, other children?
  - Have you ever had any concerns about their weight?
  - Have you ever discussed it with anyone, or sought information/advice about it? If yes – who/where? Can you tell me about it?
- Did any health professionals monitor your baby’s weight since they were born?
  - If so - Who? How often?
  - Did they plot this on a growth chart?
  - Did they explain what it meant? Did it make sense to you?
  - Did you get a copy of the record?
- Has anyone ever commented on the size of your baby/toddler?
  - If yes - Who? What did they say?
  - How did you respond/feel at the time?
  - What are your thoughts about it now?

**F. Possible interventions**

- Do you remember if any health professionals ever discussed any health messages/health-related behaviours with you while you were pregnant or since your baby was born?
  - [Open Q first => cue cards if not forthcoming]
  - The amount of weight you gained during your pregnancy
  - Your diet/what you ate during your pregnancy
  - How physically active you were during you r pregnancy
  - Feeding your baby – breastfeeding, formula feeding, introducing solid foods
  - Active play/tummy time with your baby
  - Time spent watching TV or on phones
  - Your baby’s sleep] with you? Can you tell me a little bit more about this? Who? What? What did you think about this?
- Who are the main health professionals you have contact with regarding your child (since pregnancy)?
  - To what extent do you seek information/advice from them about your child’s weight/growth?
  - Was the advice you got useful? Did you take it on board (why/why not)?
  - How would describe your relationship with them?
  - To what extent do you trust them?
  - How does this compare to other sources of information/advice (i.e. family, friends, social media)?
- What other practitioners do you see? Where else do you go for advice/information?
- What do you think works to encourage healthy growth?
  - How do you think we can encourage healthy growth in babies and toddlers?
  - Where did these ideas come from?
  - What would you find useful? / What have you found useful in the past?
- How/when do you think healthy growth in young children can be promoted, and who do you think can do that?

[Note: wording in this section to be adapted for interviews with fathers – e.g. ‘your pregnancy’ -> ‘the pregnancy’, ‘with you’ -> ‘with you or your partner/the mother of your baby’.]

**G. Summary / Close**

- [Note to self: Reflect back a summary of what was asked during the interview and what they said]
- Was there anything I left out?
- Is there anything you would like to add?
