# Supplementary material for: “They Just Need to Come Down a Little Bit to Your Level”: A Qualitative Study of Parents’ Views and Experiences of Early Life Interventions to Promote Healthy Growth and Associated Behaviours
Source: Int J Environ Res Public Health. 2020 May 21;17(10):3605. doi: 10.3390/ijerph17103605 (PMC7277501; doi:10.3390/ijerph17103605)
Supplement: Supplementary file 1 [file ijerph-17-03605-s001.zip › SupplementaryMaterials/AdditionalFile4_InformationandSupport-SourcesandTopics.docx]

**Additional File 4 Information and support: sources and topics**

Parents had a range of sources of information and support during the first 1,000 days, with health professionals, peers/family, online sources (‘Dr Google’, specific websites, online forums, email updates), fathers and ‘own research’ being the most common. Participants described a range of interactions with various health professionals, including GPs, midwives, PHNs, lactation consultants, and to a lesser extent obstetricians, pediatricians, and practice nurses. When asked, and prompted if necessary, about discussions with health professionals around child growth-related behaviours, parents primarily recalled those around infant and child feeding.

Few participants recalled any advice received around preconception issues, smoking during pregnancy, or screen-time. Discussions about a mother’s diet during pregnancy seemed infrequent and varied, if they happened at all. Sources of information included midwives (at booking visits), GPs, and antenatal classes. The focus of discussions seemed to be on food safety issues and what a woman could/couldn’t eat during pregnancy rather than ‘healthy eating’ per se. Many participants recalled discussions, however brief, about physical activity during pregnancy – with midwives and physios at antenatal classes, at booking visits and at GP visits. While information at antenatal classes seemed to focus on benefits of activity in preparation for birth, discussions with GPs centred more on asking women if they were active and encouraging them to be/maintain a certain level of exercise. Some participants noted that they had to ask their GP for advice around pregnancy physical activity and if they could maintain their pre-pregnancy levels.

There were mixed reports of discussions around gestational weight gain. Some participants reported being monitored (particularly those with an issue, e.g. gestational diabetes) by midwives/obstetricians in hospitals and/or GPs, with (e.g. told it was normal) or without discussion, while others reported that it was not discussed at all. Even when women were weighed, sometimes this was only at the initial booking visit, whereas others were weighed throughout.

Participants discussed a wide range of sources of information/support when it came to infant/child feeding topics such as breastfeeding, bottle-feeding, introducing solids (including baby led weaning). These included antenatal classes (breastfeeding), PHNs (all topics), midwives/hospital nurses and doulas (breastfeeding), with GPs mentioned to a lesser extent. Few mentioned doing ‘weaning’-specific breastfeeding classes while four also mentioned doing workshops. Websites, online forums and books/booklets (e.g. those provided by PHN) were also popular.

Infant sleep discussions were mentioned by some – often with negative reactions. Some participants reporting feeling frustrated if they were asked if their baby was sleeping. PHNs - and to a lesser extent lactation consultants, GPs and antenatal classes (with one using a sleep consultant) – were referenced most in this regard. Often discussions focused on sleep in the context of breastfeeding/safe sleep, with co-sleeping being controversial.

The majority of participants spoke about receiving information/advice around active play or tummy time (primarily the latter) from PHNs though hospital midwives, physiotherapists at discharge/postnatal class, GPs, lactation consultants, antenatal classes, and family/peers were mentioned by a small number of participants.
